# Supplementary material for: Within-Host Adaptation of Staphylococcus aureus in a Bovine Mastitis Infection Is Associated with Increased Cytotoxicity
Source: Int J Mol Sci. 2021 Aug 17;22(16):8840. doi: 10.3390/ijms22168840 (PMC8396210; doi:10.3390/ijms22168840)
Supplement: Supplementary file 1 [file ijms-22-08840-s001.zip › ijms-1283014-supplementary.pdf]

International Journal of *Molecular Sciences*

MDPI

Supplementary Materials:

Within-Host Adaptation of *Staphylococcus Aureus* in A Bovine Mastitis Infection Is Associated with Increased Cytotoxicity

Katharina Mayer [1], Martin Kucklick [2,3], Helene Marbach [1], Monika Ehling-Schulz [1], Susanne Engelmann [2,3], and Tom Grunert [1,*]

[1] Functional Microbiology, Institute of Microbiology, Department of Pathobiology, University of Veterinary Medicine, 1210 Vienna, Austria; katharina.mayer@vetmeduni.ac.at (K.M.); helene.marbach@vetmeduni.ac.at (H.M.); monika.ehling-schulz@vetmeduni.ac.at (M.E-S.)

[2] Institute for Microbiology, University of Technical Sciences, 38106 Braunschweig, Germany; martin.kucklick@helmholtz-hzi.de (M.K.); susanne.engelmann@helmholtz-hzi.de (S.E.)

[3] Microbial Proteomics, Helmholtz Centre for Infection Research, 38124 Braunschweig, Germany

* Correspondence: tom.grunert@vetmeduni.ac.at

International Journal of
Molecular Sciences

Suppl. Figure S1

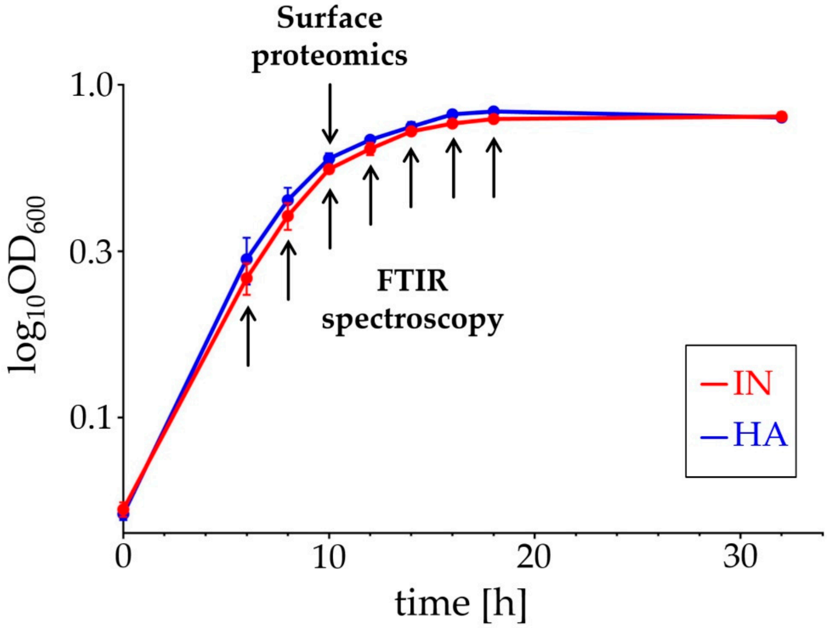

Growth curves and sampling points. IN and HA isolate were grown in iron-depleted RPMI 1640 media under oxygen-limiting conditions. Samples for surface proteomics were harvested in the early stationary phase after 10 h. Samples for the FTIR spectroscopic analysis to decipher changes in the *S. aureus* glycopolymer composition were harvested between 6 to 18 h every two hours.

International Journal of *Molecular Sciences*

MDPI

Suppl. Figure S2

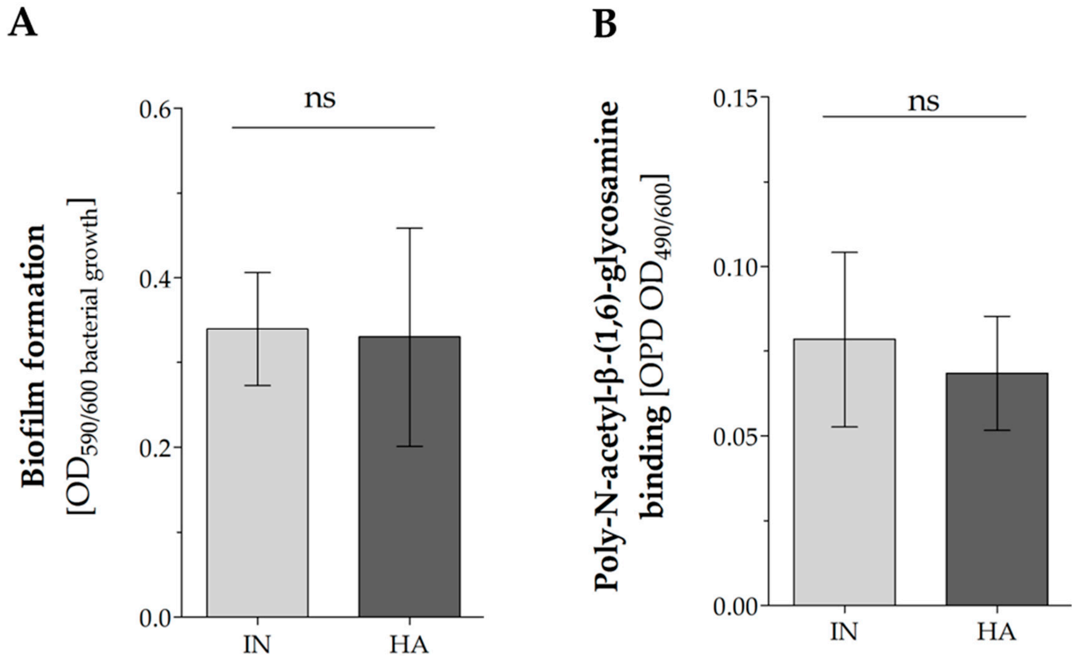

Biofilm formation and PNAG production in RPMI for 24 h at 37°C. A. The data shown represent the amount of biofilm stained by crystal violet (OD 590 nm) normalised to the bacterial growth (OD 600 nm). **B.** Data shown are the results of bacterial PNAG detected by WGA–HRP (OD 490 nm). (A, B) Both graphs show the mean and 95 % CI of three independent experiments, each performed as six technical replicates. IN, initial isolate; HA, host-adapted isolate.

Suppl. Table S1: Results of MS-based surface proteomics detected in significantly different quantities in the IN and HA isolates

Protein annotation[a]	EMBL ID[a]	Regulation HA[b]	Shaving - Ratio Sum Intensity (HA/IN)[c]	Biotin- Ratio HA/IN (SILAC)[c]	Gene[d]	Location[e]
Hypothetical protein Newbould305_0930	EJE57358.1	DOWN	0.222; p<0.001	n.d.	-	Lipid anchored
Alkaline shock protein 23	EJE56063.1	DOWN	0.072; p<0.001	0.062	*asp23*	Intracellular / membrane-associated
HPr kinase/phosphorylase	EJE56542.1	UP	Detected in HA only	n.d.	*hprK*	Intracellular
Ribonucleotide-diphosphate reductase subunit beta	EJE56510.1	UP	1.55	Detected in HA only	*nrdF*	Intracellular/ predicted trans-membrane
Gluconate operon transcriptional repressor	EJE56795.1	DOWN	n.d.	0.074	*gntR*	Intracellular
Hypothetical protein Newbould305_1521	EJE56769.1	DOWN	0.109	n.d.	-	-

[a] According to reference strain *S. aureus* Newbould 305

[b] UP/DOWN: Significant fold change calculated by Perseus or LFQ intensity difference of at least 4-fold between HA and IN and intensity $>2x10^6$

[c] n.d.= not detected

[d] According to reference strain *S. aureus* SACOL

[e] Localisation based on references of AureoWiki and Locate P

Suppl. Material and Methods: Detailed description of MS-based surface proteomics

Preparation of surface-associated protein fractions

1. Bacterial growth

Bacterial isolates were grown under iron- and oxygen limiting conditions in RPMI 1640 media, earlier described to be best-mimicking growth in vivo during mastitis [11]. Bacteria were cultivated overnight in RPMI 1640 medium supplemented with 0.15 mM deferoxamine (DFOM), 12C-L- Leucine, and either 12C-L- Lysine and L-Arginine (Sigma-Aldrich, St. Louis, Missouri, United States) or 13C-labeled L-Lysine and L-Arginine (used as internal standard) (Silantes; Munich, Germany). Main cultivation was started with OD_{600} 0.05 in the same medium. For the proteomics approaches, strains were harvested at 10 h (early stationary phase) in three independent experiments.

2. Surface-shaving approach

Bacterial surface-shaving was performed as described by Dreisbach *et al.* 2010 [63] with some modifications. An aliquot of 25 µl immobilised trypsin treated with L-1-tosylamido-2-phenylethyl chloromethyl ketone (TPCK) (Thermo Scientific, Pierce, Rockford, IL, USA) was resuspended in 100 mL 50 mM ammonium bicarbonate (Sigma-Aldrich) for activation, pelleted by centrifugation (370 x g, 5 min) and, subsequently washed twice with PBS supplemented with 40 % sucrose and 20 mM azide ('buffer 1') (Sigma-Aldrich). The pelletised trypsin and cell pellets obtained from 3 ml bacteria culture at OD_{600} 0.2 were resuspended in 50 µl 'buffer 1' and incubated for 45 min at 37°C to shave proteins and soluble domains of proteins at the bacteria cell surface. After centrifugation (6000 x *g*, 10 min), shaved protein domains in the supernatant were reduced with 10 mM DTT (Serva, Heidelberg, Germany) and alkylated with 10 mM iodoacetamide (Sigma) (each reaction for 30 min in the dark) and subsequently digested with 20 ng trypsin (Promega, Madison, Wisconsin, United States) overnight at 37°C. The enzyme reaction was stopped by acidification using 2 µl 10 % formic acid (FA) (Fisher, Honeywell-Fluka, Schwerte, Germany). Peptides were desalted with C18-ZipTips (Merck Millipore, Burlington, Massachusetts, United States), which were equilibrated stepwise with 20 µl acetonitrile (ACN) 80% ACN 0.1% FA, 50% ACN 0.1% FA, 30% ACN 0.1% FA and 0.1% FA before 20 µl of the sample was aspirated ten times to bind the peptides on the C_{18} material of the tips.

Purification of the peptides was carried out by washing four times with 20 µl 1% FA, followed by the elution with 20 µl 50% ACN 0.1% (4x) and FA 80% ACN 0.1% FA (4x). This procedure was performed with two aliquots of each sample, which were pooled afterwards. In the end, the sample volume was reduced to 13 µl by vacuum centrifugation (Eppendorf concentrator plus, Eppendorf, Hamburg, Germany).

1.2 Surface-biotinylation approach

The bacterial surface-biotinylation approach was performed as described by Hempel *et al.*[31] with some modifications. Cell pellets of 90 ml bacteria culture at OD_{600} 0.4 were resuspended in 1 ml PBS (pH8) supplemented with 1 mM Phenylmethylsulfonylfuorid (PMSF) (Carl Roth GmbH, Karlsruhe, Germany) and mixed with 100 µl 1% Sulfo-NHS-SS-Biotin solution (Pierce Biotechnology, Rockford, IL, USA). Next, the mixture was incubated for two hours by gently shaking (50 rpm/min) on ice in the dark. The cells were centrifuged (4000x *g*, 5 min, 4°C) and washed three times with ice-cold PBS (pH8) supplemented with 500 mM glycine (Carl Roth). For cell disruption, cell pellets were resolved in 500 µl PBS (pH8) supplemented with 1 mM PMSF and transferred into ribolyzer tubes (Carl Roth) filled with 0.5 mL of 0.1 mm glass beads (Carl Roth). Mechanical cell disruption was conducted in a FastPrep homogeniser (MP Biomedicals, Solon, OH, USA) at 6 m/s2 for 20 sec two times. Cell lysates were centrifuged (20,000x *g*, 30 min, 4°C), and cell pellets were resuspended in 500 µl PBS supplemented with 1% NP-40 ('buffer 2') (Thermo Fisher Scientific). For NeutrAvidin agarose affinity-purification (Thermo Fisher Scientific), 50 µl agarose-beads were washed twice in buffer 2, centrifuged (1000x *g*, 1 min, 4°C) and resuspended in 50 µl 'buffer 2'. Samples were incubated with the agarose beads for 90 min by shaking gently on ice in the dark. The NeutrAvidin agarose resins were centrifuged and washed six times with 'buffer 2'.

Elution of peptides was conducted by a reductive SDS sample buffer, composed of 62.5 mM Tris, 2 % SDS, 20 % glycerol, 50 mM DTT and 5 % beta-mercaptoethanol (pH6.8) (Merck, Darmstadt, Germany). The biotin-binding agarose beads were incubated with 20 µl SDS buffer for 5 min by gently shaking and 5 min by fast shaking (125 rpm/min). After that, samples were centrifuged (50x *g*, 1 min) two times and transferred to a new tube each time to remove all the NeutrAvidin agarose resins.

Next, the solution of the biotinylated proteins and a SILAC standard (four biological replicates of IN and HA cultivated under the same conditions in medium suppl. with ^{13}C L-Lysine and L-Arginine; Silantes; Munich, Germany) were mixed 1:2, loaded onto a SDS-PAGE and further processed as described in Toyofuku *et al.*[64] using a modified digestion buffer (50 mM TRIS/HCl, 1 mM $CaCl_2$*)* and an additional extraction step with ACN. For in-gel digestion, each lane was cut into eight subsamples of similar protein amounts and pooled with a second lane of the same biological replicate to increase the protein concentration. Peptide desalting was done by using C18-ZipTips (Merck Millipore). Samples were vacuum dried in a speed vac and stored at -20°C.

1.3 LC-MS/MS analysis

For liquid chromatography-tandem mass spectrometry (LC-MS/MS) analysis, a nanoAQUITY Ultra Performance Liquid Chromatography System (Waters Corporation, Milford, MA, USA) was coupled to an LTQ Orbitrap Velos Pro mass spectrometer (Thermo Fisher Scientific Inc). Peptides from each sample fraction were solved in 3% acetonitrile and 0.1%

formic acid, centrifuged for 20 min at 109,000x *g* and loaded onto a BEH C18 column, 130 Å, 75 µmx 250mm at a flow rate of 0.35 ml/min (Waters Corporation). Elution of peptides from the column was performed using a 205 min gradient starting with 3.7% buffer B (80% acetonitrile and 0.1% formic acid), and 96.3% buffer A (0.1% formic acid in Ultra-LC–MS water): 0–30 min 3.7% B; 30–65 min 3.7–22.1% B; 65–88 min 22.1–29.3% B; 8–148 min 29.3–48.3% B; 148–175 min 48.3–62.5% B; 175–192 min 62.5–99% B; 192–195 min 99% B; 195–200 min 99–3.7% B, 200–205 min 3.7% B.

Primary MS scans were performed in the Fourier transformation mode, scanning an m/z of 350–1,900 with a resolution (full width at half maximum at m/z 400) of 60,000 and a lock mass of 445.12003. Primary ions were fragmented in a data-dependent collision-induced dissociation mode for the 20 most abundant precursor ions with an exclusion time of 12 s and analysed by the LTQ ion trap. The following ionization parameters were applied: normalized collision energy: 35, activation Q: 0.25, activation time: 10 ms, isolation width: 2m/z, charge state: 2 to 4. The signal to noise threshold was set to 2,000.

The MS/MS data of three biological replicates were analysed by MaxQuant (version 1.5.2.8) and Perseus (version 1.4.1.3) software (Max-Planck-Institute of Biochemistry, Martinsried, Germany). For protein identification and quantification (by MaxQuant), the following settings were used: label-free quantification (surface shaving) or sample multiplicity: 2 with unlabeled and 13C labelled arginine and lysine (biotinylation), a match between runs, peptide tolerance: 5 ppm; tolerance for fragment ions: 0.6 Da, fixed modification: carboxyamidomethylation of cysteine, variable modifications: oxidation of methionine, biotinylation, *Staphylococcus aureus* (ST97, RF122, N305) databases from NCBI (downloaded 2017-05-30). A maximum of three modifications per peptide was allowed; the fixed false discovery rate (FDR) was set to 1% for peptide spectrum matches, peptides and proteins.

MS data filtering and statistical analyses were done using the proteinGroups.txt output file of MaxQuant. Proteins with a minimum of two unique peptides detected by at least one MS/MS scan in two different samples or replicates were considered for reliable identifications. A label-free quantification method using LFQ intensities (surface shaving) or a label-based method using a labelled reference protein sample (super-SILAC, biotinylation) was applied for protein quantification. If the sum of raw intensities (or light intensities in super-SILAC) of an identified protein for the three replicates was ≤ 500.000, LFQ intensities were set to zero or the proteins were filtered out (in super-SILAC).

A Post hoc analysis was performed with the Perseus software. For statistical analyses, LFQ intensities or light/heavy quotients were transformed into log2 values and standardised using Z-score. A permutation FDR-based Students t-test was applied to filter proteins with statistically significantly different amounts. Additionally, the data set was evaluated manually to identify proteins that can be only detected in one of the *S. aureus* isolates. Essential information of genes incl. protein subcellular location was retrieved using Aureowiki and LocateP [65,66].
